# Hard polymeric porous microneedles on stretchable substrate for transdermal drug delivery

**DOI:** 10.1038/s41598-022-05912-6

**Published:** 2022-02-03

**Authors:** Aydin Sadeqi, Gita Kiaee, Wenxin Zeng, Hojatollah Rezaei Nejad, Sameer Sonkusale

**Affiliations:** 1 Nano Lab, Advanced Technology Laboratory, Tufts University, 200 Boston Avenue, Medford, MA 02155 USA; 2 Department of Electrical and Computer Engineering, Tufts University, 161 College Ave, Medford, MA 02155 USA; 3 Anodyne Nanotech Inc, 38 Wareham St, Boston, MA 02118 USA

**Keywords:** Engineering, Biomedical engineering, Drug delivery

## Abstract

Microneedles offer a convenient transdermal delivery route with potential for long term sustained release of drugs. However current microneedle technologies may not have the mechanical properties for reliable and stable penetration (e.g. hydrogel microneedles). Moreover, it is also challenging to realize microneedle arrays with large size and high flexibility. There is also an inherent upper limit to the amount and kind of drugs that can be loaded in the microneedles. In this paper, we present a new class of polymeric porous microneedles made from biocompatible and photo-curable resin that address these challenges. The microneedles are unique in their ability to load solid drug formulation in concentrated form. We demonstrate the loading and release of solid formulation of anesthetic and non-steroidal anti-inflammatory drugs, namely Lidocaine and Ibuprofen. Paper also demonstrates realization of large area (6 × 20 cm^2^) flexible and stretchable microneedle patches capable of drug delivery on any body part. Penetration studies were performed in an ex vivo porcine model supplemented through rigorous compression tests to ensure the robustness and rigidity of the microneedles. Detailed release profiles of the microneedle patches were shown in an in vitro skin model. Results show promise for large area transdermal delivery of solid drug formulations using these porous microneedles.

## Introduction

Microneedles have been developed as an effective method for transdermal drug delivery^1–4^. It has received attention since it avoids degradation of the drugs in the gastrointestinal tract and bypasses the first-pass effects associated with the liver in case of oral delivery, and the pain and inconvenience of intravenous injection through the skin^3,5–7^. Also using microneedles offers a minimally invasive, less painful and self-administrable approach for drug delivery. Researchers have proposed different manufacturing methods for microneedles with applications in drug delivery, wearables and implantables^8–11^. UV lithography^12–18^, drawing lithography^19–21^, deep X-ray lithography of Lithographie Galvanoformung Abformung (LIGA)^22,23^, micromilling^24,25^, Deep Reactive Ion Etching (DRIE)^26–31^, wet etch technology^32^, 2D and 3D printing^33–39^ have been proposed for their fabrication. However these methods have an inherent limitation in terms of cost and complexity of fabrication making microneedles an expensive solution compared to ingestibles or subcutaneous injections. There is a need for a fabrication process which is more cost-effective and requires less time to make them   in a flexible format. Besides the different methods of fabrication, there are different kinds of microneedles categorized as solid^40^, hollow^41,42^, dissolving^43–45^, merged-tip^46^ and porous^47–54^ microneedles. Solid microneedles can be made from metals or bio-compatible and stiff polymers. It can be used for creating pores in the outer layers of the skin and then applying the drug topically. Since pores can heal rapidly, these microneedles cannot be used for long term sustained release. Another method of using solid microneedles is to coat the drug on the surface of the solid microneedle arrays^55–59^. The coating would dissolve, releasing the drug transdermally in the skin tissue. One of the disadvantages of this approach is the low quantities of drug that can be coated onto the needles. Solid microneedles also have safety issues for shattering while inside the skin becoming a biohazard. Hollow microneedles have microfluidic channels that carry the drug from a separate reservoir into the skin. They provide a mechanism to hold large quantities of drug but the process to make them is complicated requiring cleanroom processes, while there are drawbacks that only drugs in liquid form can be delivered^42^. Dissolving microneedles patches contain drugs in hydrogel or bio-resorbable polymers and are generally safe compared to solid microneedles where there is potential for bio-hazard from disposal of microneedles. Dissolving microneedles are made from several bio-absorbable polymers like maltose^60^, sugar^61^, salmon sperm DNA (SDNA)^62^, poly(methylvinylether maleic anhydride) (PMVE/MA)^63^, carboxymethyl cellulose (CMC)^43,64^ and polyvinylpyrrolidone (PVP)^65^. One of the drawbacks is that these unwanted polymers (non-drug) are also released along with the drug and there is a limit on the quantity of the drug that can be delivered. These microneedles are also less reliable for skin penetration and breakage. Porous microneedles are another alternative which carry a large volume of distributed drug loaded pores. The surface porosity of the microneedles carries the potential to deliver large quantities of drug. However it is challenging to make hard porous microneedles that can penetrate skin reliably. There are different approaches to make porous microneedles utilizing different materials like silicon^66,67^, colloidal silica^68^, polymers^69^, ceramics^70^, gypsum brushite^52^, alumina^50,71^ and metal^54^. In some cases these microneedles are made partially with porous material such as having only a porous tip^48^. Microneedles with a porous polymer have also been made using polymerization in the presence of a porogen (pore template)^72^. Although the synthesized polymer had high mechanical strength, the resultant microneedles had inconsistent geometry, which made them unsuitable for further applications. Poly lactic acid (PLA) porous microneedles have been used for transdermal drug delivery but these microneedles also lack the strength and are not able to penetrate to skin^49^. We need the microneedles to be mechanically rigid, and sufficiently sharp to enable easy penetration into the skin. Moreover, the base substrate should be flexible enough to conformably adapt to the curvature and deformations of the human body to improve wearer comfort, facilitate effective penetration and sustained release of drug into skin over wear-time. Finally, we expect the microneedles to be available both in small and large formats to meet different dosing requirements for different parts of the body.

In this paper, we introduce a new kind of polymeric porous microneedle that uses direct solid drug formulation embedded in a UV-curable biocompatible hard polymer. Drugs are distributed in the pores of these solid and hard microneedles. Use of solid drug formulation allows one to load large quantities of drug compared to aqueous dispersions of drugs. It also expands the kind and type of drugs that can be loaded. We performed compression tests on these microneedles and showed that they are robust from breakage on skin penetration while providing excellent and reliable penetration in the epidermis. For proof of concept, these new class of porous microneedles were used to deliver two important drugs, namely Lidocaine and Ibuprofen. Lidocaine is a popular local anesthetic and Ibuprofen is a non-steroidal anti-inflammatory drug (NSAIDs) used for pain relief. Injecting hypodermic needles for delivering Lidocaine and Ibuprofen is painful, it can cause trypanophobia, hypersensitivity, bruising and bleeding^73^. Oral administration does not target the pain area and has a longer time to act. Also, it can have significant adverse effects, namely gastrointestinal (GI), renal, and cardiovascular damage^74^. Topical creams or sprays containing local anesthetics have been developed as a means to avoid needle injections^75–79^. The use of topical anesthetics is limited due to their slow onset and short duration. Targeted delivery of Lidocaine and Ibuprofen using microneedles can provide rapid relief. Also, it has a higher efficacy compared to topical patches.

In this paper, we made both small and large area porous microneedle patches with flexible backing for local pain relief that can be applied on any body part (even elbow or knee) with excellent conformability. Drug release profile of Lidocaine and Ibuprofen patches has been measured in vitro. Also, we verified the efficacy of Lidocaine and Ibuprofen drugs after embedding in the polymeric resin matrix of the microneedles. Our research confirms that Lidocaine and Ibuprofen maintained their molecular structure after being formulated with resin. The mechanical behavior of the microneedles was also investigated by histological examination and through compression tests with excellent results as discussed below.

## Results

### Polymeric porous microneedles

**Figure 1:**
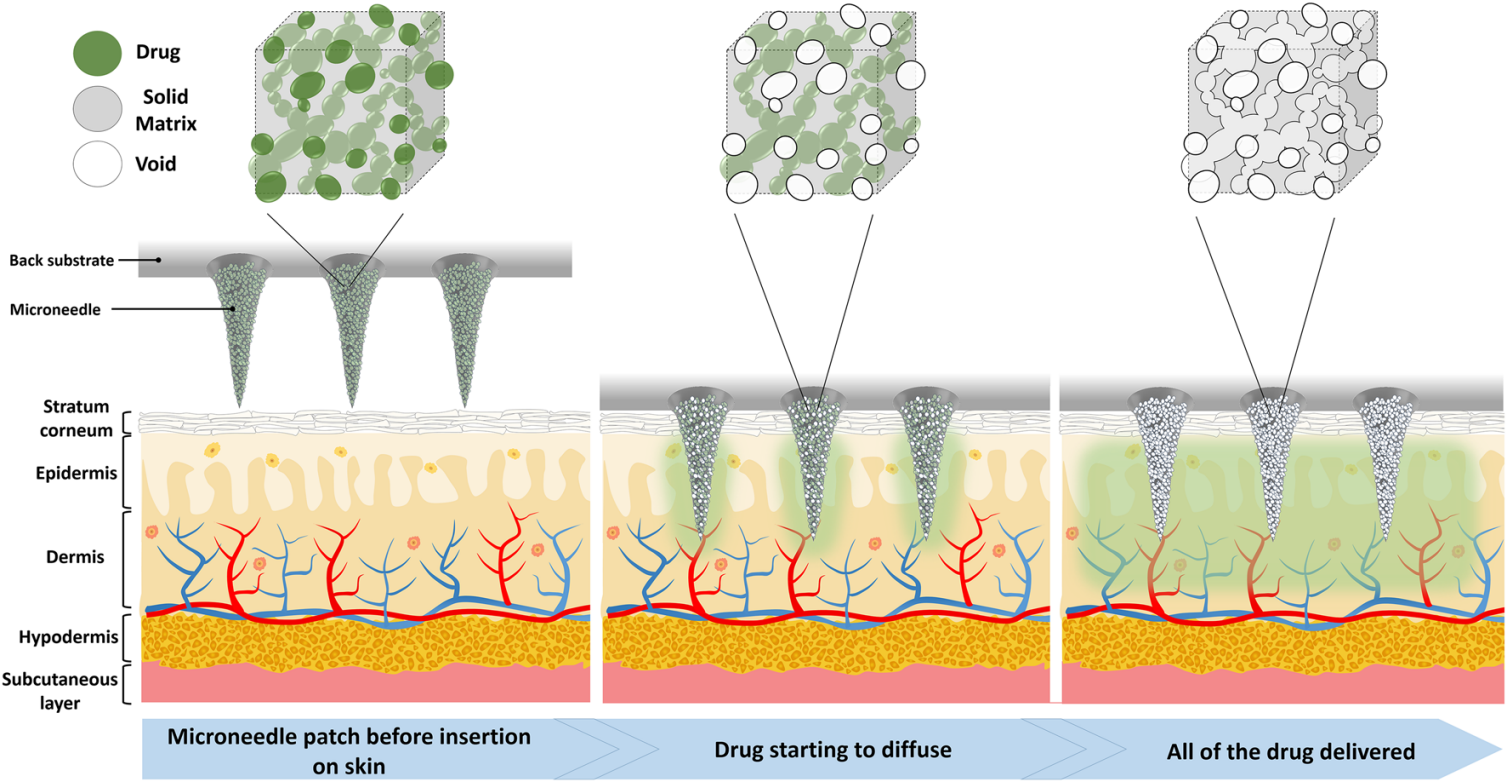
Schematics of the polymeric porous microneedles with solid drugs embedded inside the solid matrix before insertion into skin, after insertion into skin and after sustained release.

Our proposed polymeric porous microneedles are made by casting a colloidal mixture of the solid drug powder and a biocompatible/photo-curable resin. Having a solid drug formulation ensures the highest possible concentration of the drug can be loaded into the microneedles. The resin creates a solid matrix around the network of drug powder particles which are organized as pores and microchannels (see Fig. 1 for conceptual representation) in a hard and solid resin. After insertion into the skin, the drug particles dissolve in the interstitial fluid of the skin tissue diffusing out leaving behind a network of open channels and voids as shown in Fig. 1. The rigidity of the resin maintains the mechanical strength of the microneedles during penetration without getting damaged or fractured.

### Microneedle fabrication procedure

**Figure 2:**
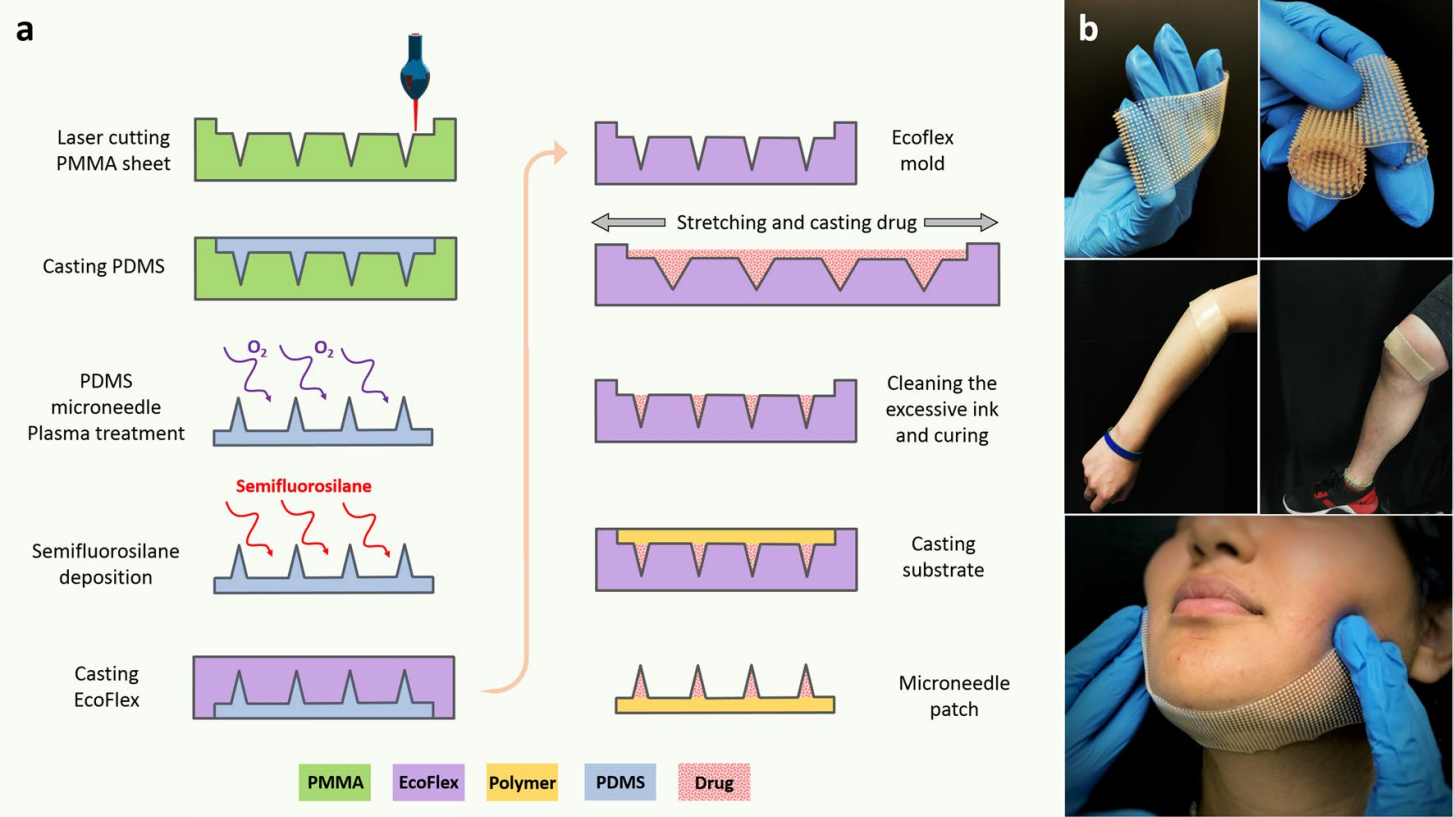
(**a**) Fabrication flow of the microneedle mold and microneedle patch, (**b**) patches of microneedle showing the flexibility, conformality and a roll of the microneedle patch.

Any microneedle mold could be used to cast the drug/resin formulation to make microneedle patches. However, we chose to use our prior work on cross-over-lines (COL) fabrication procedure^34^ to make microneedle patches due to its low cost, easy processing and ability to make large area patches, as shown in Fig. 2a. A CO_2_ laser (Boss LS-1416 from Boss Laser, LLC; Sanford, FL, USA) was used to create negative volume on a Clear Scratch- and UV-Resistant Cast Acrylic sheet (part number of 8560K359. McMaster-Carr; Princeton, NJ, USA). The engraved acrylic mold was washed with isopropanol and distilled water to remove the dust and particles from the surface and engraved areas. A nitrogen gun was used to remove the excess water on the surface. The mold was then dried in an atmospheric oven at 80 °C for 30 min. Then, polydimethylsiloxane (PDMS, Dow Sylgard™ 184 Silicone Elastomer; Dow Silicones Corporation in Midland, MI, USA) was cast on the acrylic sheet. The PDMS-casted sheet was degassed and subsequently cured in the oven at 80 °C for 2 h. After complete curing of the PDMS, PDMS microneedles were peeled off of the acrylic sheet and were treated with oxygen plasma to activate the surface of the PDMS microneedles. The PDMS microneedles were then silanized with trichloro(1H,1H,2H,2H-perfluorooctyl) silane (SKU: 448931-10G, from MilliporeSigma; Burlington, MA, USA) under vacuum in a desiccator overnight. Ecoflex™ 00–50 (Smooth-On, Incorporated, Macungie, PA, USA) with the ratio of 1:1 was cast on the silanized PDMS microneedles followed by curing at room temperature. The silane layer creates a barrier between PDMS microneedles and Ecoflex mold, avoiding them from bonding to each other, and facilitates their detachment. The final Ecoflex mold can be used to create microneedles by casting different polymers. In our case, the drug solution or powder can be cast on the stretched mold. In our study we introduced a new drug/resin paste composed of biocompatible/photo-curable resin and the solid drug powder, explained in the earlier section. Since the viscous dough-like drug paste could not be embedded directly in the tiny holes of microneedle molds by regular vacuum molding or centrifugal methods, we optimized our previously-proposed fabrication method^34^ for microneedles fabrication by using the stretchable polymer for microneedle molds. Having a stretchable microneedle mold enabled us to cast highly-viscous drug/resin paste into the microneedle molds through mechanical blade coating of the paste onto a stretched mold surface. Also, utilizing the stretchable mold decreases the microneedle fabrication time drastically by eliminating the vacuum procedure to embed the drug solution in micron-size cavities of microneedle molds. After molding the drug/resin paste onto a stretched Ecoflex microneedle mold, the mold was then released from stretch causing excessive drug paste to accumulate on the top, this is then removed from the surface of the mold ensuring drug/resin formulation is limited to the tip geometry of the microneedle. The paste is then cured through exposure to 405 nm UV light. The biocompatible resin used to fabricate the microneedles results in a polymeric matrix with hard mechanical properties after curing. After the curing step, a thin layer of elastic polymer is cast on the surface of the mold as a substrate and cured under UV light. Using a hard polymer for the needles and elastic polymer for the substrate enables us to have stiff and rigid microneedles that facilitate effective insertion into the skin tissue and a soft/elastic substrate which enables skin conformability and stretchability. Finally, the microneedles with the substrate were peeled off the mold with needles bonded to the back substrate. As shown in Fig. 2b, we made several patches including the large area 6 × 20 cm^2^ microneedle patch and demonstrated its mechanical properties. These flexible microneedle patches with hard microneedles can be fabricated in any form and shape by following the procedure mentioned above. The resulting microneedles are porous microneedles with drug powder distributed within the pores and the micro-channels of the microneedles.

### Dye-loaded microneedle patch for in vitro release

**Figure 3:**
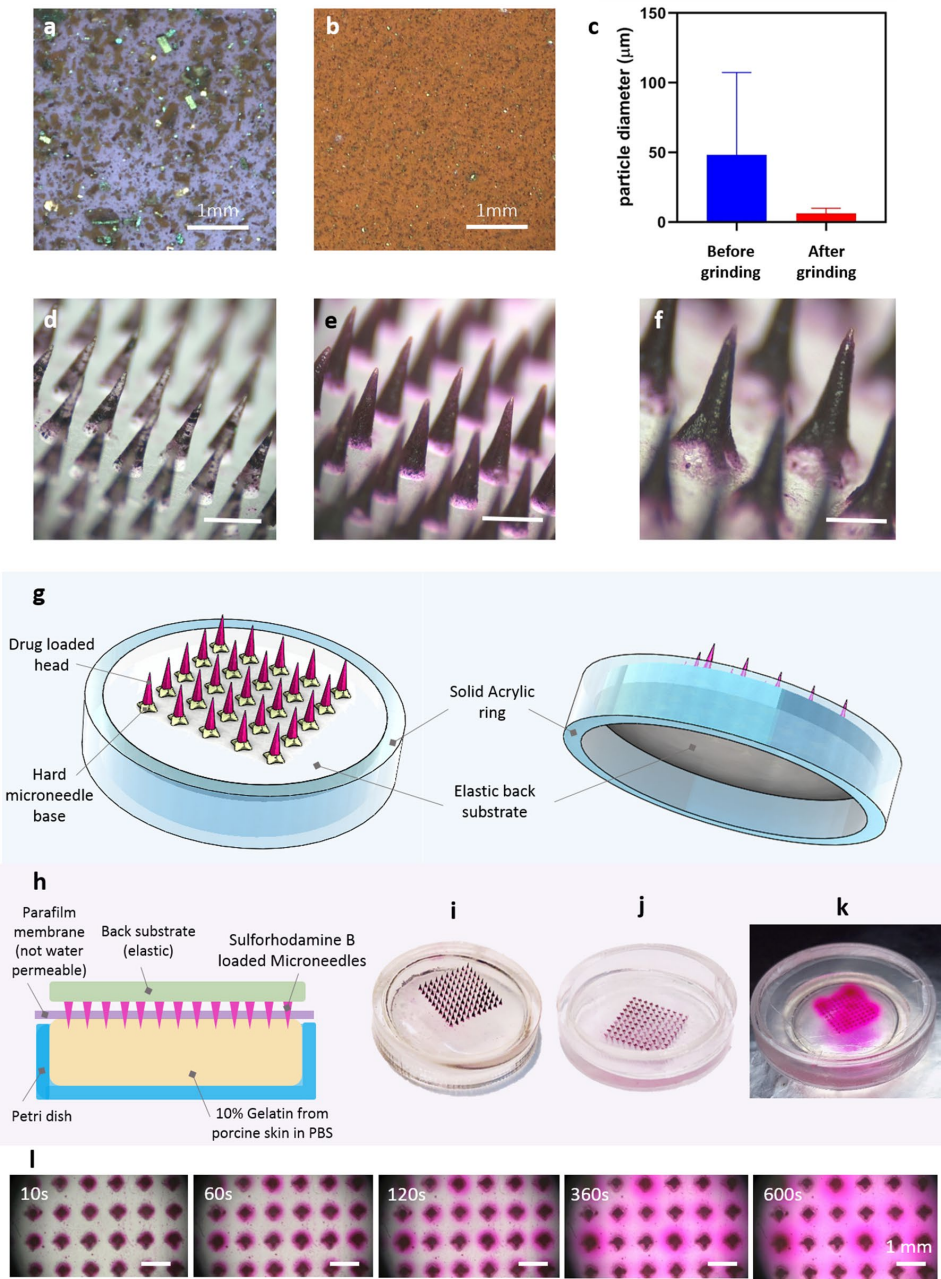
(**a**) Unground Sulforhodamine B particles, (**b**) ground Sulforhodamine B particles, (**c**) particle size distribution of Sulforhodamine B particles before and after grinding, (**d**) microneedles made with unground Sulforhodamine B particles, scale bar of 1 mm, (**e**) microneedles made with ground Sulforhodamine B particles, scale bar of 1 mm, (**f**) microneedles made with ground Sulforhodamine B particles with base structure, scale bar of 0.5 mm, (**g**) schematics of microneedle arrays with elastic back substrate and solid acrylic ring, (**h**) in vitro release experiment schematics, (**i**) front side of dye-loaded microneedle patch, (**j**) back side of dye-loaded microneedle patch, (**k**) release of dye in gelatin after 10 min, (**l**) release distribution of dye-loaded microneedle arrays in gelatin at different time stamps.

In order to show qualitative in vitro release of the microneedles visually, we first used a colored dye as a model drug for easier visualization. We used bio-compatible/photo-curable resin (Dental SG) from Formlabs (Somerville, MA, USA) and Sulforhodamine B (SKU: 230162-5G, MilliporeSigma; Burlington, MA, USA). Due to the small size of microneedles, the dye particles need to be ground into finer particles with smaller sizes. Without this step, the dye particles will not distribute uniformly especially on the tips, given the tips are merely ~ 20 µm in size. We compared the encapsulation of the dye in microneedles by preparing microneedles with both unground and ground Sulforhodamine B particles. The unground and ground particles of the dye are shown in Fig. 3a,b with size distribution shown in Fig. 3c. On average, the unground and ground dye particles had ~ 50 µm and ~ 6 µm size respectively. As shown in Fig. 3d, there is a lesser quantity of the dye particles encapsulated due to larger size. On the other hand, the microneedles shown in Fig. 3e have more dye particles encapsulated. Figure 3f shows dye-loaded microneedles with star-shaped base structure which can be added for robustness and reliable attachment of the microneedles to the base utilizing the same COL process to make the molds^34^. Note that the dye does not dissolve in the resin but gets dispersed in the resin. Mixing the resin and the drug for longer times will result in more even distribution. Also, homogenization and sonication would result in a more even distribution. Figure 3g shows the schematics of the microneedle patch that we prepared for in vitro dye release. We added a solid acrylic ring around the substrate so that the patch can be held and applied easier on the skin. The in vitro dye release experiment is shown schematically in Fig. 3h. A 10% gelatin (Gelatin from Porcine Skin, SKU: G2500-1KG from Millipore Sigma) solution was prepared as a tissue model and was poured onto a petri dish. Then it was placed in the refrigerator for 20 min for solidification. A thin layer of parafilm (Parafilm^®^ M, Amcor; Zurich, Switzerland) was then used as an outer stratum corneum skin layer to cover the gelatin tissue model in the petri dish. The top and bottom side of the prepared dye-loaded microneedle patch is shown in Fig. 3i,j. After inserting the microneedle patch into the “parafilm-covered solidified-gelatin” skin model, the dye gets released into the gelatin which is visually monitored at time points as shown in Fig. 3l. Figure 3k shows the release of the dye after 10 min. Results indicate that the dye loaded porous microneedle exhibits sustained release in gelatin, making this type of microneedle attractive for transdermal release of several drugs directly in solid form.

### Drug-loaded microneedles

The microneedle patches were loaded with an anesthetic drug, Lidocaine and a NSAID Ibuprofen. We studied the in vitro drug diffusion profile for both of these drugs. We also confirmed if the molecular structure of the drug and thus its efficacy is maintained throughout the process of making the microneedles. We performed FTIR spectroscopy on the cured drug/resin for this confirmation. We also analyzed the mechanical properties of drug-loaded microneedles through compression tests.

### In vitro drug release of lidocaine and ibuprofen microneedles

**Figure 4:**
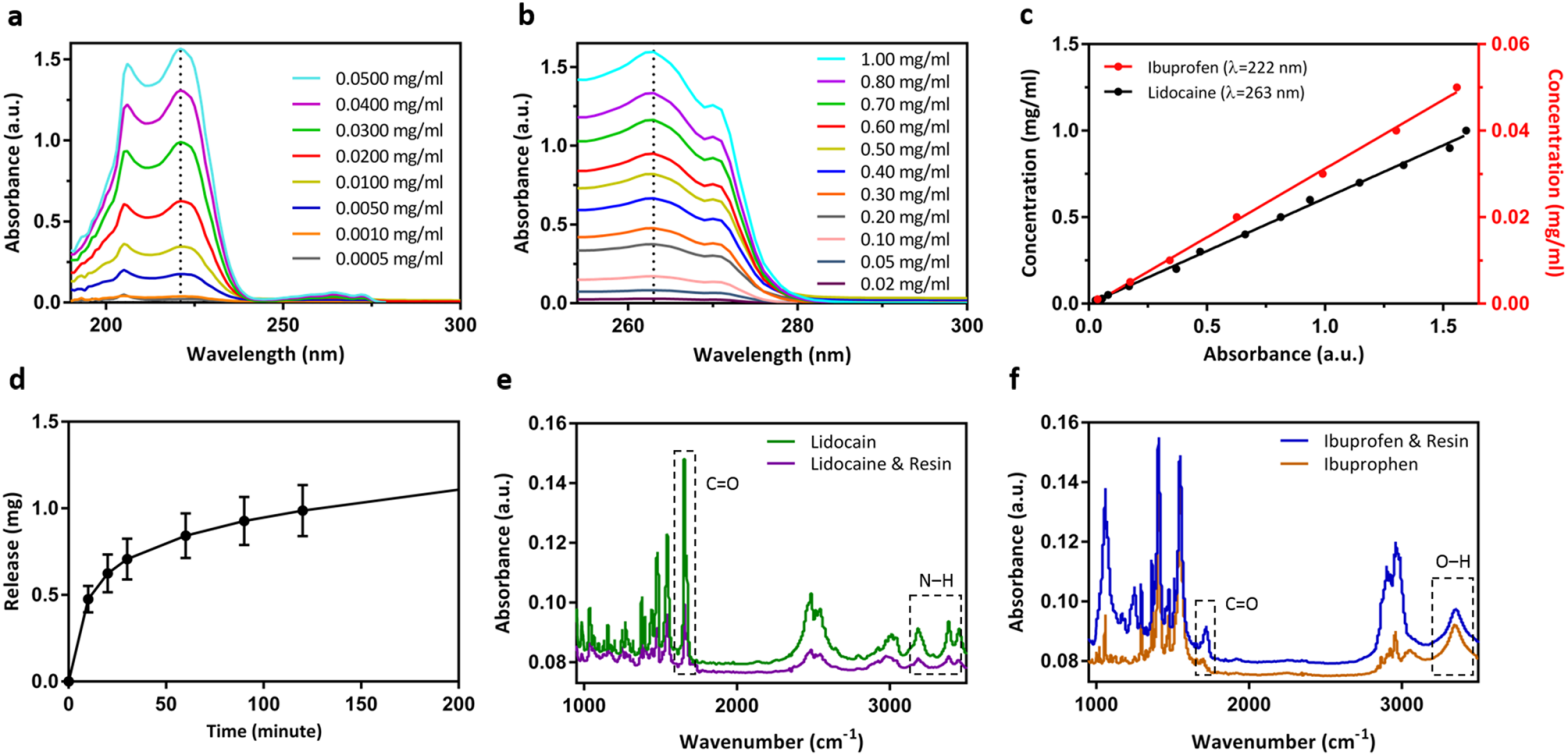
(**a**) UV–Vis spectroscopy of Ibuprofen for different concentrations in mg/ml, (**b**) UV–Vis spectroscopy of Lidocaine for different concentrations in mg/ml, (**c**) calibration curve of Ibuprofen and Lidocaine showing absorption amplitude in 222 nm and 263 nm for different concentrations, (**d**) release profile of Ibuprofen/resin and Lidocaine/resin microneedle patches in DPBS, each patch had one hundred microneedles, (**e**) FTIR spectroscopy of Lidocaine encapsulated in microneedle showing absorbance spectrum of lidocaine powder compared with Lidocaine/resin, (**f**) FTIR spectroscopy of ibuprofen encapsulated in microneedle showing absorbance spectrum of Ibuprofen powder compared with ibuprofen/resin.

We used Ibuprofen sodium salt (SKU: I1892) and Lidocaine hydrochloride monohydrate (SKU: L5647) both purchased from MilliporeSigma (Burlington, MA, USA). Ibuprofen and Lidocaine can be detected by UV–Vis spectroscopy in the 222 and 263 nm band respectively^80,81^. Different concentrations of Ibuprofen and Lidocaine were prepared by dissolving them in Dulbecco’s Phosphate Buffered Saline (DPBS) by MilliporeSigma (SKU: 59331C). The absorbance peaks at 222 nm and 263 nm for Ibuprofen and Lidocaine solutions were detected by Evolution 220 UV–Vis Spectrophotometer by Thermo Fisher Scientific Incorporated (Waltham, MA, USA). The absorption spectrums were swept in 190–300 nm and 254–300 nm of wavelength for various concentrations as shown respectively in Fig. 4a,b. We extracted the calibration curve for Ibuprofen and Lidocaine based on the data points of the absorbance amplitude versus concentration (mg/ml) as seen in Fig. 4c. The calibration curve helps us to quantify the release profile of the drug in the DPBS medium. We performed a detailed release study in DPBS medium for both Ibuprofen and Lidocaine microneedle patches. The Ibuprofen sodium salt and Lidocaine hydrochloride monohydrate were ground by Chulux grinder (with four blades) for three minutes to make finer particles. Then, Ibuprofen and Lidocaine fine particles were mixed with bio-compatible/photo-curable resin with a weight ratio of 1:1. After that, the patch was fabricated with the same method explained in the “Microneedle Mold Fabrication Procedure” section. Each patch had one hundred microneedles on it with a size of 0.5 mm in diameter and 1 mm in height. Multiple petri dishes (one petri dish for each time stamp) containing DPBS were placed inside the Midi CO_2_ incubator (Thermo Scientific) with a temperature of 37 °C. As shown in Fig. 4d, the release profile shows that each patch (containing 100 microneedles) roughly releases 1 mg of drug in DPBS solution after 2 h by dissolution-diffusion mechanism. Considering that some drug powders are encapsulated in the areas close to the base and the core of microneedles, it would take more time for those drug powders to come out of the solid matrix and in some cases some drug powders might choose to stay entrapped in the solid matrix. This would happen for lower pore densities. Therefore one can increase the porosity such that almost all of the drug would release given sufficient time. For the microneedles reported in this publication with 1:1 resin:drug ratio, our calculations indicate that around 20% of the drugs gets released after 200 min of study. Increasing the amount of resin and decreasing the amount of drug will reduce the amount of drug encapsulation reducing our overall drug capacity. On the other hand, decreasing the amount of resin and increasing the amount of drug powder would result in a weaker structure for the needles and consequently lower breaking force for the needles.

### Interaction between polymers and drug: FTIR spectroscopy

FTIR spectroscopy was used to evaluate the possible changes in encapsulated drugs during the casting and curing process. Our expectation is that the drugs do not change their form and molecular structure during mixing and UV curing. We used Nicolet 6700 (Thermo Scientific™; Waltham, MA, USA) with a Smart™ iTX ATR accessory having a diamond crystal to acquire Fourier transform infrared attenuated total reflectance (FTIR-ATR) spectra. The spectra showed the presence of the following characteristic peaks in lidocaine: N–H stretching at 3450 and 3385 cm^−1^, amide C=O stretching at 1655 cm^−1^ as shown in Fig. 4e. There was an obvious increase in the intensity of the peak at ∼1655 cm^−1^, related to the amide C=O stretch. In FTIR, an increase in the peak intensity usually means an increase in the amount (per unit volume) of the functional group associated with the molecular bond, whereas a shift in peak position usually means the hybridization state or electron distribution in the molecular bond has changed. Thus, the decrease in the intensity of amide C=O in lidocaine/resin samples was attributed to the reduction of lidocaine ratio in samples^82^. The FTIR was checked with a manufacturer data sheet for Lidocaine hydrochloride monohydrate and there were three peaks at 3450, 3400 and 3200 cm^−1^ as seen in our data in Fig. 4e. Also, no shift in peak position was seen in FTIR spectra of ibuprofen/resin as seen in Fig. 4f. The peaks at 1721 cm^−1^ and 3400 cm^−1^ are assigned to the stretching vibration of C=O and O–H, respectively^83^. FTIR observation confirmed that the chemical structure of lidocaine and ibuprofen remained unchanged during the fabrication process.

### Histology test, surface morphology and mechanical behavior

**Figure 5:**
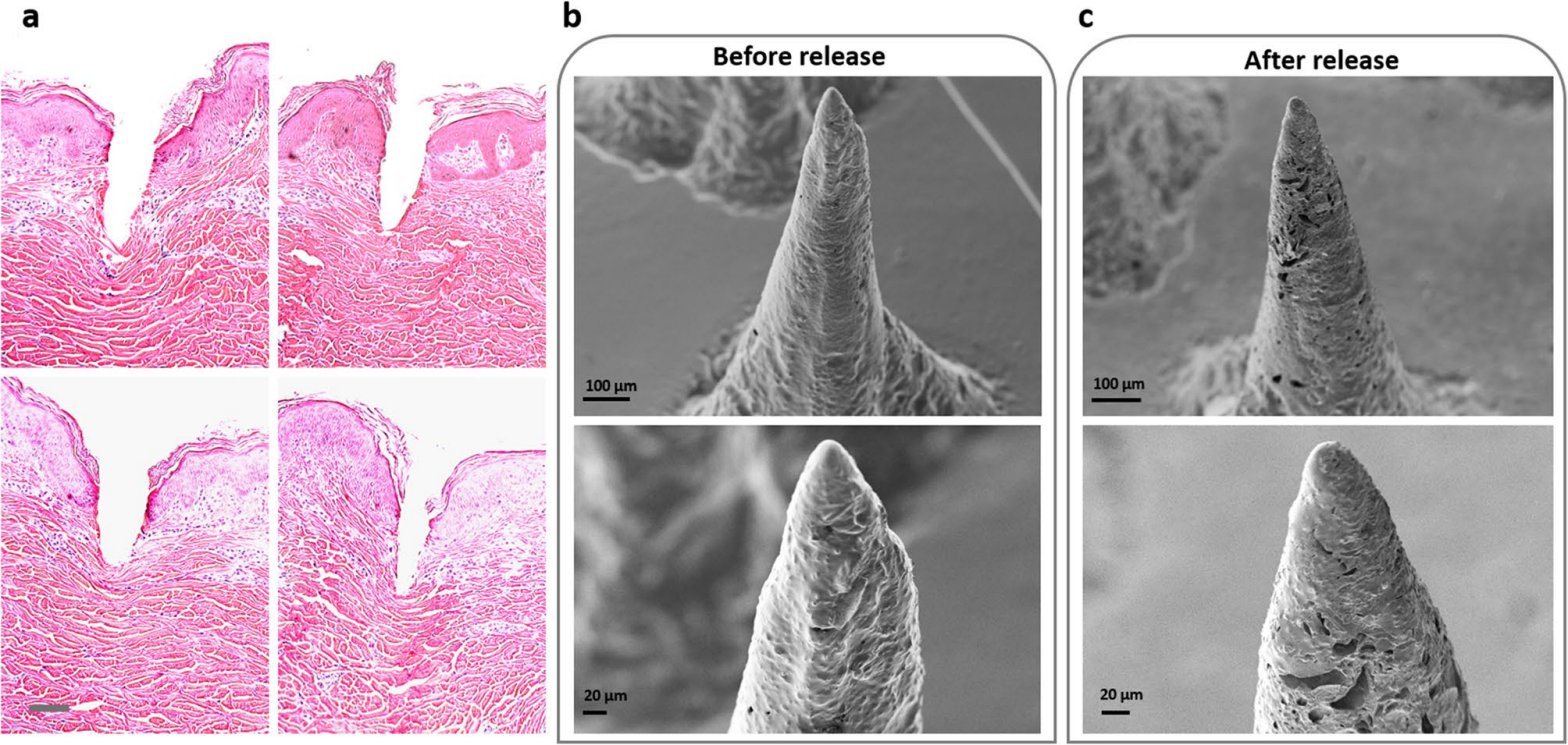
(**a**) Histology examination of the microneedles, scale bar 100 µm, (**b**) SEM images of resin/drug before release, (**c**) SEM images of resin/drug after release.

A histology test was performed to confirm the insertion of microneedles to the skin. A pig skin from a recently sacrificed four month old male Yorkshire pig (from another study) was used for the histology test. The skin was shaved and cut using a 10# scalpel blade and was placed into a specimen container filled with sterile 0.9% saline. The microneedle patch was inserted on the skin using a thumb pressure. The micrographs are from the H&E (Hematoxylin and Eosin) stained tissue section fixed in 10% neutral formalin. Histological examination showed that the microneedle penetrated ~ 600 µm onto the skin as shown in Fig. 5a. As it was shown in other similar studies, the depth of microneedle’s penetration into skin was shorter than the length of the entire microneedle due to the deformation of highly elastic skin^43,84,85^. Having an optimal length of microneedles or penetration depth is a tradeoff between numerous variables like desired drug delivery dosage, microneedle application site, spacing, numbers and diameter of MNs, velocity of microneedle application etc.

Next we studied the pore structures in the microneedles. The mixture of resin and drug creates pores shown in Fig. 5b. As discussed before, these pores are created in a solid matrix. The drug gets released after administering the microneedles into the skin, leaving empty cavities behind. These empty cavities are observable in Fig. 5c which are SEM images of the microneedles after release.

**Figure 6:**
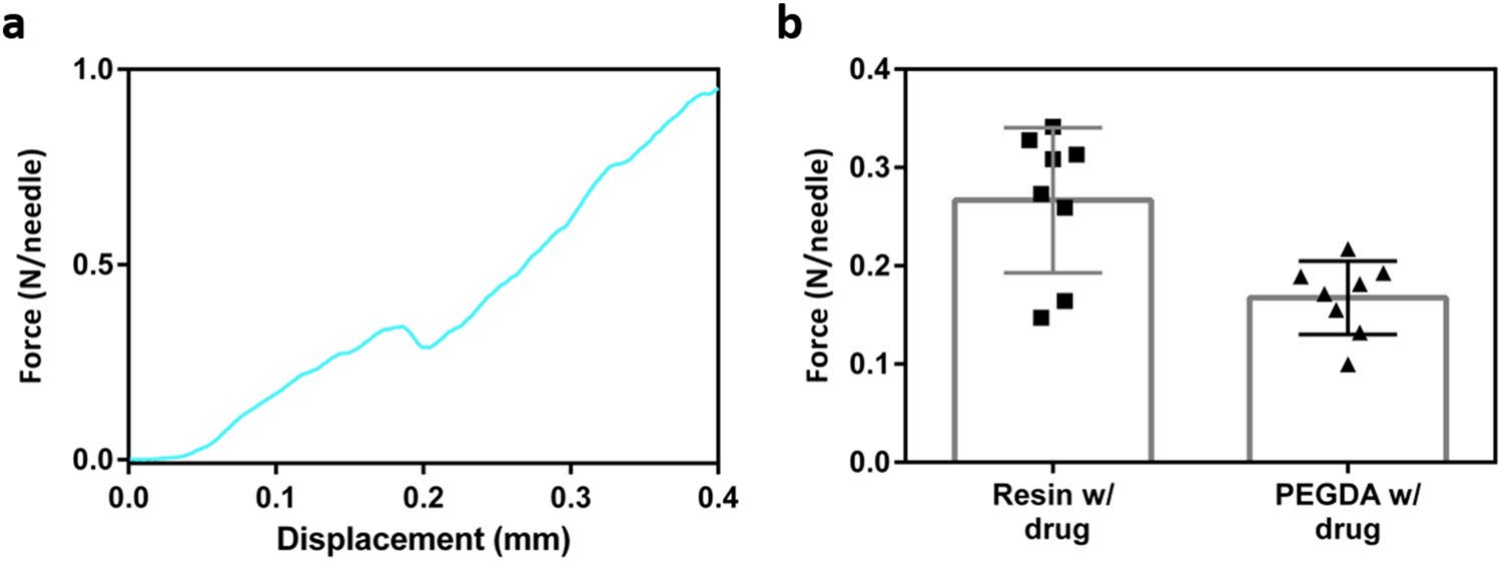
(**a**) Mechanical behavior of an individual microneedle containing drug and resin, (**b**) comparing the robustness of resin/drug microneedle with PEGDA/drug microneedle.

Our porous microneedles showed high robustness due to high tensile strength of the resin (73 MPa). An individual drug/resin microneedle showed a mechanical behavior as displayed in Fig. 6a. The compression test was performed with Instron (Norwood, MA, USA). The microneedles tip started to break down at the force of ~ 0.26 N per needle on average. This is over four times the required force needed for insertion into the skin using the microneedles of this geometry^12^.

As a final set of experiments, we compared drug/resin microneedles used in this study to another representative of a hard microneedle made from polyethylene glycol diacrylate (PEGDA) based microneedles. The PEGDA microneedles also show strong mechanical properties for easy skin penetration as detailed in previous studies^86^. In our study, we confirmed higher robustness of drug/resin microneedles compared to the PEGDA based microneedles as shown in Fig. 6b. The PEGDA microneedles were prepared with a Lidocaine solution of 500 mg/ml and PEGDA (Millipore Sigma, SKU: 437441-500ML) with molecular weight of 575 mixed 1% photo initiator of 2-Hydroxy-4′-(2-hydroxyethoxy)-2-methylpropiophenone (Millipore Sigma, SKU: 410896-10G). The drug solution and PEGDA solution was mixed with the ratio of 4:1 (v/v). The prepared solution was added to microneedle mold and crosslinked by UV-light irradiation with a wavelength of 365 nm. Our results indicate that PEGDA based microneedles are less rigid and they start to break down at the force of ~ 0.164 N per needle.

## Discussion

In this paper we presented a new kind of polymeric porous microneedles through direct mixing of a solid drug with biocompatible and photo-curable resin. Solid formulation provides an opportunity to increase drug loading capacity in the microneedles compared to aqueous drug dispersions. Our polymeric microporous microneedles have reliable mechanical properties for effective skin penetration. The microneedle patches have flexible and stretchable backing that improves skin conformability and wearability. Patches can be made large or small and in any shape and form to be compatible with application at different body parts. To demonstrate drug release, Lidocaine and Ibuprofen were embedded in our proposed porous microneedles with an eye towards local pain management. We investigated their release profile in DPBS and verified the drugs characteristics are unchanged after mixing and curing them with the polymers. We also confirmed the ex vivo penetration of the microneedles in pig skin by histology examination which showed desirable penetration behavior. Solid drug formulation ensures that different drug candidates, both large and small molecules, can be delivered at high strengths using these microneedles as compared to conventional microneedles.
